# Age is no factor in TAVI

**DOI:** 10.1007/s12471-022-01725-4

**Published:** 2022-09-02

**Authors:** R. Adrichem, N. M. Van Mieghem

**Affiliations:** Department of Interventional Cardiology, Thoraxcenter, Erasmus University Medical Center, Rotterdam, The Netherlands

Transcatheter aortic valve implantation (TAVI) is constantly evolving and improving as treatment option for severe aortic stenosis. To date, TAVI has proven to be at least comparable to surgical aortic valve replacement (SAVR) in elderly patients across the entire operative risk spectrum [1].

Randomised controlled trials (RCTs) comparing TAVI with SAVR in patients with severe aortic stenosis at very high, high and intermediate risk for early mortality after surgery typically revolved around octogenarians. In contrast, recent low-risk RCTs featured a distinct age creep to a mean age < 75 years [1]. American and European Cardiology guidelines on valvular heart disease responded by introducing an age reference denoting proper candidates for TAVI (> 65 years in the US and > 75 years in Europe), regardless of other disease states/morbidities/frailty. Interestingly, no upper age limit was suggested above which valve therapy should be deferred.

Over the last decade, an aging society and overall rise in life expectancy of men and women caused an increase in the relative proportion of elderly patients. Another important clinical observation is that over the years, the phenotype of elderly patients has drastically changed. These days, elderly patients not only add years to life, but also add life to years. A change which postulates a paradigm shift in the approach of the elderly patient with aortic stenosis.

The first two landmark TAVI RCTs evaluated patients who were either deemed inoperable (PARTNER 1 Cohort B [2]), or operable, but at high operative risk (PARTNER 1 Cohort A [3]). If anything, inoperable patients were slightly younger, but the calculated surgical risk score was similar for both patient cohorts (STS-PROM > 11%). Inoperable patients benefitted from a dramatic 20% absolute reduction in all-cause mortality at 1 year (only 5 patients needed to be treated to save 1 life), high-risk patients showed similar survival out to 5 years after TAVI or SAVR. Regrettably, these RCTs also showed that approximately 50% of patients did not benefit from the TAVI procedure because they either had died, or had not experienced any significant improvement in quality of life after 1 year [4].

This futility paradigm remains highly relevant. Historically, advanced age has been a principle argument to turn down patients from open heart surgery [5], and a major contributor to established risk score models such as the EUROSCORE II and STS-PROM. Arguably, SAVR leaves a higher physical impact and implies a longer recovery than TAVI. The undisputed less invasive nature of TAVI begs the question whether age is as relevant in predicting procedural outcome after TAVI. Even more so because conventional risk scores have been validated and calibrated in surgical cohorts and have performed notoriously poor in TAVI.

Clearly, risk scores or age alone cannot distinguish patients who may benefit from TAVI. Apart from medical history and comorbidities, the concept of frailty appeared to be an important discriminator for TAVI “responders”. Frailty is characterised by compromised energetics, neurocognitive impairment, and progressive dependency in daily activities. The pace of decline, however, is highly variable and person-specific to the extent that elderly patients may be living independently even late in their nineties. A multi-parametric frailty assessment therefore seems mandatory for all (elderly) patients who are considered for TAVI and justifies active participation of geriatricians in contemporary multi-disciplinary heart valve teams.

The paper by Van den Brink et al. in this issue of the Netherlands Heart Journal reinforces the notion that age by itself should not discriminate eligible patients for TAVI [6]. This single-centre retrospective analysis reported similar 30-day and 1‑year all-cause mortality after TAVI in patients ≤ 85 years (U85) and patients > 85 years (O85), (4.4% vs 5.6% and 6.6% vs 5.6%; *p* = 0.521). Notably, baseline characteristics and calculated risk scores were remarkably similar between cohorts apart from mean age (78.5 years vs 87.3 years respectively). Still, taking into consideration that age remains an important variable in the EUROSCORE II, a similar EUROSCORE II suggests that the younger cohort had more comorbidities and may have been more frail. Indeed, the U85 cohort had numerically more COPD, previous CABG and prior aortic valve surgery, and previous stroke, which may explain the paradoxically longer hospital stay in these patients (6.29 days vs 5.98 days; *p* = 0.037).

Other registries that evaluated TAVI in the very elderly showed conflicting results. Vendrik et al. showed comparable mortality in patients above and below 90 years old up to five years [7]. Conversely, Vlastra et al. and Murthi et al. reported an increase in in-hospital mortality for patients > 90 years and > 80 years respectively [8, 9]. An analysis from the STS/ACC TVT registry showed increased 1-year mortality of nonagenarians compared with younger patients, although STS-PROM scores were higher in the older patient cohort and the observed-to-expected ratios of mortality remained on par in the very elderly [10].

Collectively, these registries suggest that advanced age should not necessarily be a determining factor to decline aortic valve treatment. In fact, meticulous patient selection is key to minimise futility in elderly patients with a plethora of phenotypes.

Finally, attention in the Netherlands should now shift to the lower-risk spectrum of patients with aortic stenosis. Two large RCTs confirmed early safety benefits with TAVI over SAVR in low-risk patients with a mean age of 74 years. Comparative trials with follow-up out to 5 and 8 years with different transcatheter valve platforms reported similar outcomes and at least equal (sometimes even better) bioprosthetic valve performance in the mid-term after TAVI [1]. This growing body of evidence is reassuring and should be communicated to the patient with symptomatic severe aortic stenosis for a balanced shared decision-making, a concept that is endorsed by all major cardiology guidelines. Today, also in the Netherlands, every single aortic stenosis patient who is set to receive an aortic bioprosthesis (not a mechanical prosthesis) should get the option to consider the less invasive treatment under local anaesthesia with minimised hospital stay and expedited recovery, ambulation and return to normal life.
